# Two spurge species, *Euphorbia resinifera O. Berg* and *Euphorbia officinarum subsp. echinus (Hook.f. & Coss.) Vindt* inhibit colon cancer

**DOI:** 10.1186/s12906-024-04566-3

**Published:** 2024-07-10

**Authors:** Rania Benjamaa, Anlin Zhu, Soeun Kim, Dohyang Kim, Abdel Khalid Essamadi, Abdelkarim Moujanni, Anass Terrab, Namki Cho, Jaewoo Hong

**Affiliations:** 1 Department of Physiology, Daegu Catholic University School of Medicine, Daegu, 42472 South Korea; 2 Faculty of Sciences and Technologies, Laboratory of Biochemistry, Neurosciences, Natural Resources, and Environment, Hassan First University of Settat, Settat, 26000 Morocco; 3 CaniCatiCare Inc., Daegu, 42078 South Korea; 4 College of Pharmacy, Chonnam National University, Gwangju, 61186 South Korea; 5 Department of Plant Biology and Ecology, University of Seville, Seville, 41012 Spain

**Keywords:** *Euphorbia resinifera O. Berg*, *Euphorbia officinarum subsp. echinus (Hook. f. & Coss.) Vindt*, Colon cancer, Spurge, *Euphorbia*

## Abstract

**Background:**

Colon cancer, a prominent contributor to global cancer-related deaths, prompts the need for innovative treatment strategies. *Euphorbia resinifera O. Berg* (*E. resinifera*) and *Euphorbia officinarum subsp. echinus Hook. f. & Coss Vindt* (*E. echinus*) and their bee-derived products have been integral to traditional Moroccan medicine due to their potential health benefits. These plants have historical use in addressing various health issues, including cancer. However, their effects against colon cancer remain unclear, and the specific mechanisms underlying their anti-cancer effects lack comprehensive investigation.

**Methods:**

The study aimed to assess the potential anti-cancer effects of *Euphorbia* extract on colon cancer cell lines (DLD-1) through various techniques. The apoptosis, migration, and proliferation of DLD-1 cells were measured in DLD-1 cells. In addition, we conducted High-Performance Liquid Chromatography (HPLC) analysis to identify the profile of phenolic compounds present in the studied extracts.

**Results:**

The extracts demonstrated inhibition of colon cancer cell migration. *E. resinifera* flower and *E. echinus* stem extracts show significant anti-migratory effects. Regarding anti-proliferative activity, *E. resinifera* flower extract hindered proliferation, whereas *E. echinus* flower extract exhibited dose-dependent inhibition. Apoptosis assays revealed *E. resinifera* flower extract inducing early-stage apoptosis and *E. echinus* flower extract promoting late-stage apoptosis. While apoptotic protein expression indicated, *E. resinifera* stem and propolis extracts had minimal impact on apoptosis.

**Conclusion:**

The findings provide evidence supporting the beneficial effects of *E resinifera* and *E. echinus* extracts on colon cancer and exerting anti-cancer properties.

**Supplementary Information:**

The online version contains supplementary material available at 10.1186/s12906-024-04566-3.

## Introduction

Cancer remains a significant global health challenge, accounting for many deaths [1]. Among the causes of cancer-related mortality, metastasis accounts for approximately 90%, and its underlying mechanisms are not yet fully elucidated [2]. Metastasis begins with the dissemination of the primary tumor from its originating organ or tissue to surrounding or adjacent structures, eventually reaching distant sites through systemic or lymphatic circulation [3].

On a global scale, colon cancer ranks as the third most lethal cancer affecting both men and women, making it one of the most prevalent forms of cancer [4]. Annually, over a quarter million individuals are diagnosed with colon cancer [5]. The development of various types of colon cancer can be attributed to genomic alterations that disrupt cell cycle control, apoptosis regulation, migration, and cell proliferation pathways [6]. However, one of the most significant disadvantages of current chemotherapies against colorectal cancer is the toxic side effects [7], such as nausea, vomiting, inhibition of bone marrow function, alopecia, and cell mutations, which patients dread [8]. For this reason, many scientists and healthcare providers have been interested in medicinal plants and their derivatives to prevent or treat cancer [9]. Owing to their structural stability, the chemical structure of the compounds, and secondary metabolites like flavonoids, alkaloids, terpenoids, etc., plants and their derivatives show relatively low toxicity with high potency [4, 10, 11].

The *Euphorboaceae*, well-known as the spurge family, is one of the most prominent flowering plants, containing about 300 genera, including almost 7,500 species [12]. They grow in the form of shrubs, trees, herbs, or lianas with irritant milky latex [13]. Several *Euphorbia* species have fundamental economic importance and are widely used in traditional medicine [14].

Morocco has diverse plant wealth due to its geographical location, climate, and diverse habitats, providing it with diverse natural flora [15], which plays a more prominent economic role [16]. Indeed, traditional medicine has always occupied a significant portion of conventional Moroccan medicine [17].

*E. resinifera*, called *“Zakkoum* in Arabic, is an endemic species of Morocco, found in the south of the Mid-Atlas, particularly in the Central High Atlas area, on the side of Beni Mellal and Azilal [18]. *E. echinus*) is an endemic species of North Africa in Morocco located in the southwestern region [19], locally known as” *Daghmous*” [20].

The most studied part of these plants is the latex, which has been investigated in chemical composition. Its pharmacological and biological properties have been known as antibacterial, antifungal, and antioxidant [21–27].

Despite the importance of these two species, the pharmacological activities are still unclear, and few studies have characterized the roles of extracts from different parts of these two plants. As part of the development and valorization of Moroccan aromatic and medicinal plants, two species of *Euphorbia* have been selected based on their uses in traditional medicine.

In the current study, we initially examined the anti-cancer activity of stems, flower petals, and propolis ethanolic extract of two Moroccan *Euphorbia* species, *E. resinifera*, and *E. echinus*, on human colon adenocarcinoma cell line, DLD-1. Our study is the first comparative study to evaluate the effect on the mechanism of anti-cancer activity of two plants belonging to the same family and their hive products.

## Methods

### The collection of the plant

**Fig. 1:**
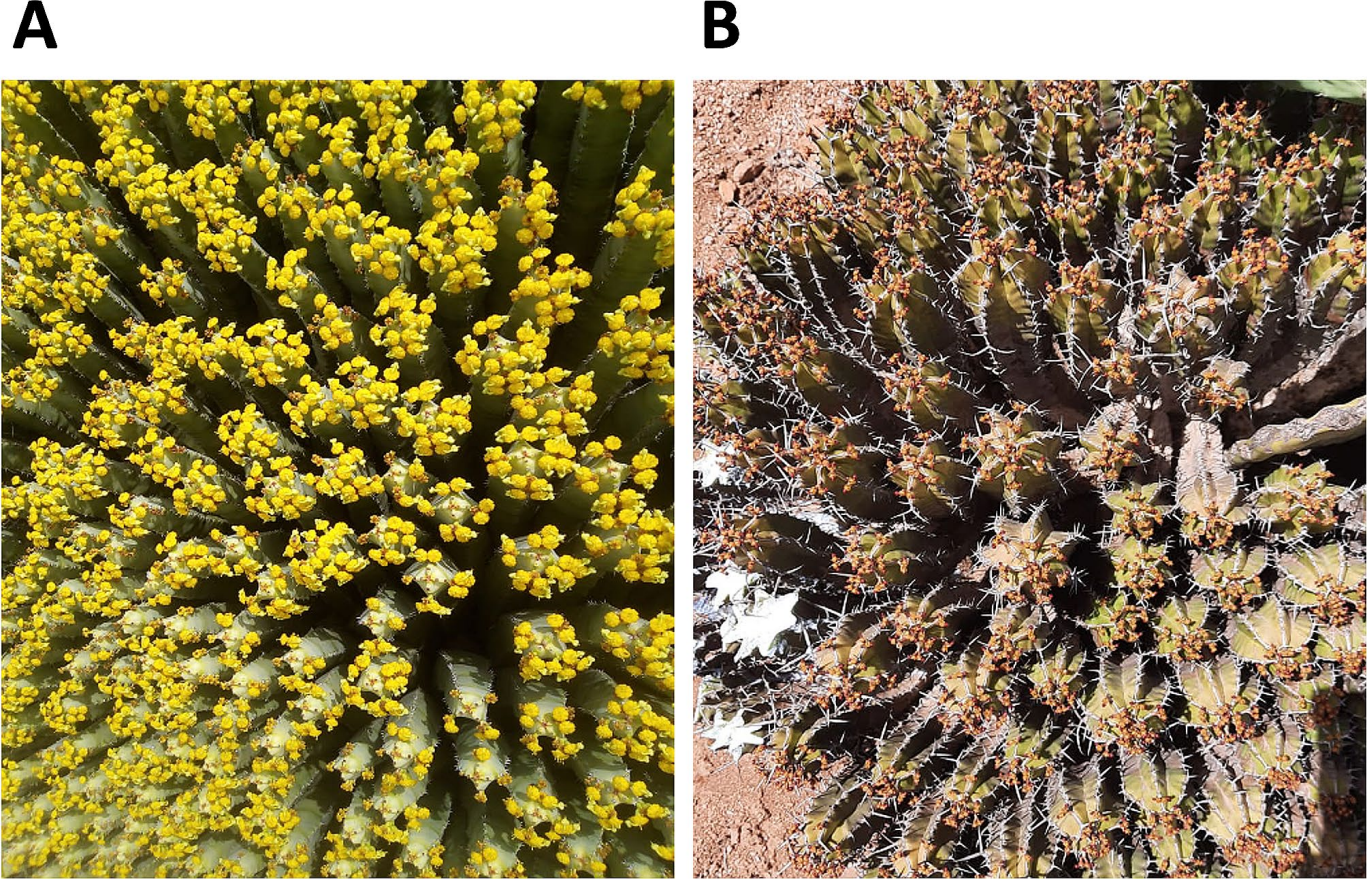
Photos of wild *Euphorbia* plants in Morocco. **(A)** *Euphorbia. resinifera* from Ouaouizeght region. **(B)** *Euphorbiaoflicinarum L. subsp. echinus* from Tiznit region

The aerial parts of *Euphorbia resinifera O.Berg* (*E. resinifera*) and *Euphorbia officinarum subsp. echinus (Hook. f. & Coss.) Vindt* (*E. echinus*), i.e., stems and flower petals, were collected from the fields of Ouaouizeght (harvest location according to Merchich northern Morocco X: 410,804 Y: 179 306) in July and Tiznit (harvest location according to Merchich Sahara nord X: 778 847 Y: 770 997) in August, respectively, in Morocco during their flowering season in 2020 under the control of the High Commission for Water and Forests in two regions (Fig. 1). The wild plants were identified at the Scientific Institute - Department of Botany in Rabat, Morocco, the voucher reference codes for *E. resinifera* is RAB114129, and for *E. echinus* is RAB114130. The propolis was collected directly from the hives of the bee farms to avoid contamination of samples. After collection, samples were kept ambient.

### HPLC analysis and purification

The dried flowers, stems, and propolis of the two *Euphorbia* species (49.6 g) were extracted using 70% ethanol with ultrasonication at room temperature. After evaporating the solvent in vacuo, the total extract (23.3 g) was subjected to RP C_18_-MPLC (10–100% MeOH), which yielded 11 fractions (E1−E11). Fraction E2 (387 mg) was separated by preparative HPLC using a 250 × 21.2 mm Phenomenex Luna C_18_ column (CH_3_CN−H_2_O 1:4), which yielded compound **2** (14.2 mg) and seven subfractions (M1−M7). Compound **6** (11 mg) was further purified from subfraction M3 (23.4 mg) by preparative HPLC with a 250 × 10 mm Phenomenex Luna C_18_ column (MeOH−H_2_O, 2:3). Subfraction M5 (75.4 mg) was isolated into five subfractions (M8−M12) by preparative HPLC using 40% MeOH in H_2_O. It yielded compound **1** (17.4 mg) and compound **4** (19.7 mg). Fraction E3 (319.8 mg) was subjected to RP C_18_-HPLC (CH_3_CN−H_2_O 3:7), which yielded eight subfractions (M13−M20). Compound **3** (4.9 mg) was obtained from subfraction M17 (9.4 mg) by preparative HPLC (MeOH: H_2_O = 9:11). Subfraction M19 (23.6 mg) was purified using preparative HPLC with 45% MeOH, which yielded compound **5** (20.5 mg).

### Cells and culture conditions

DLD-1, human colon adenocarcinoma cells, and FHC cells were obtained from ATCC (Manassas, VA). Cells were grown in Dulbecco’s Modified Eagle’s Medium (DMEM) (Welgene, Korea) supplemented with **5%** FBS and penicillin/streptomycin antibiotics. The medium was renewed every three days. Cells were maintained at 37 °C in a humidified incubator with 5% CO_2_.

### Cell viability assay

The FHC and DLD-1 cells were placed in 96-well culture plates (5 × 10^3^ cells/well). Once the cells reached confluence, they were treated with spurge extracts. After 16 h, seeding cells, a vehicle (DMSO), or different concentrations of extracts from flowers, propolis, or stem petals. These extracts were obtained from *E. resinifera* and *E. echinus* and were used at concentrations of 0, 1, 10, and 50 µg/ml. This treatment lasted for 24 h.

To assess the viability of the cells, the Quanti-MAX WST-8 Cell Viability Assay Kit reagent (Biomax, Korea) was added to the media. The mixture was then incubated at 37 °C for 30 min. After incubation, the absorbance of the solution was measured with a microplate reader at 450 nm.

### Cell migration assay

DLD-1 cells (1.10^5^ /well) were seeded in 24-well plates 24 h before the cell migration assay. Then, the cells were scratched with 200 µl pipette tips. The crude extracts at different concentrations (vehicle, 1, and 10 µg/ml) were treated and then incubated. The migration of cells was observed at 0, 24, and 48 h after incubation. The data were analyzed using DP2-BSW software [28]. The following formula calculated the migration rate in %.

$$\begin{gathered}Migration{\text{ }}rate{\text{ }}\left( \% \right)\, = \, \hfill \\\left( {Open{\text{ }}area{\text{ }}at{\text{ }}time{\text{ }}0\, - \,Open{\text{ }}area{\text{ }}at{\text{ }}the{\text{ }}measured{\text{ }}time} \right)\, \hfill \\/\,Open{\text{ }}area{\text{ }}at{\text{ }}time{\text{ }}0\, \times \,100 \hfill \\ \end{gathered}$$

### Cell proliferation assay

Anti-proliferative effects of the different extracts of *Euphorbia* were measured with Carboxyfluorescein succinimidyl ester (CFSE) staining (Invitrogen, Waltham, MA).

Briefly, CFSE-stained DLD-1 cells were seeded at 1 × 10^5^ cells per well in a 12-well plate and incubated overnight to allow adherence. Cells were incubated for 24 h with test samples at final concentrations of 1, 10, and 50 µg/ml and then trypsinized and inactivated. After washing twice with 4 °C PBS, the cell pellets were suspended in 500 µl ice-cold PBS at a density of 1 × 10^6^ cells/ml and immediately analyzed by flow cytometry to measure the fluorescence.

### Apoptosis measurement

Quantification of selective extract-induced apoptosis in DLD-1 cells was evaluated through flow cytometry using FITC Annexin V Apoptosis Detectionapoptosis kit [29] according to the manufacturer’s instructions (BD Biosciences, Franklin Lakes, NJ). DLD-1 Cells were seeded in 12-well plates at a density of 1 × 10^5^ /well and treated with indicated extract with different concentrations at 50, 10, and 1 µg/ml for 24 h. As a positive control of apoptosis, Osimertinib was treated at 0.1 µM and 1 µM concentrations. Subsequently, cells were harvested and washed twice with 4 °C PBS (Biowest, France), and then the cell pellets were suspended in 100 mL ice-cold 1X binding buffer at a density of nearly 1 × 10^6^ cells/ml and then incubated with 5 µl PE-Annexin V, and 5 µl 7-AAD for 10 min at room temperature in avoidance of light. Stained cells were detected and analyzed using flow cytometry. Finally, percentages of four different populations of cells are distinguished: unlabeled viable cells, early apoptotic, late apoptotic, and necrotic.

### Western blotting

30 µg of cell lysate was subjected to Bis-Tris Gel (10%) and transferred to PVDF for western blot analysis. The membrane was blocked with a solution containing 5% non-fat dry milk and TBST for 1 h. Primary antibodies targeting Caspase 3, PARP, and BCL-2 were acquired from Cell Signaling Technology (Danvers, MA) and were applied at a 1:1,000 dilution in the blocking solution. Secondary antibodies were obtained from Jackson ImmunoResearch Labs (West Grove, PA) and were used at a 1:10,000 dilution. The western blot results were captured using the PXi Touch system (Syngene, Frederick, MD).

### Statistical analysis

All analyses were carried out in triplicates. Data were presented as mean ± SD. A *P* value of less than 0.05 was considered significant. Statistical analyses were performed by two-way ANOVA (Tukey’s multiple comparisons test) to analyze the groups’ differences.

## Results

### Composition of Euphorbia extracts

**Fig. 2:**
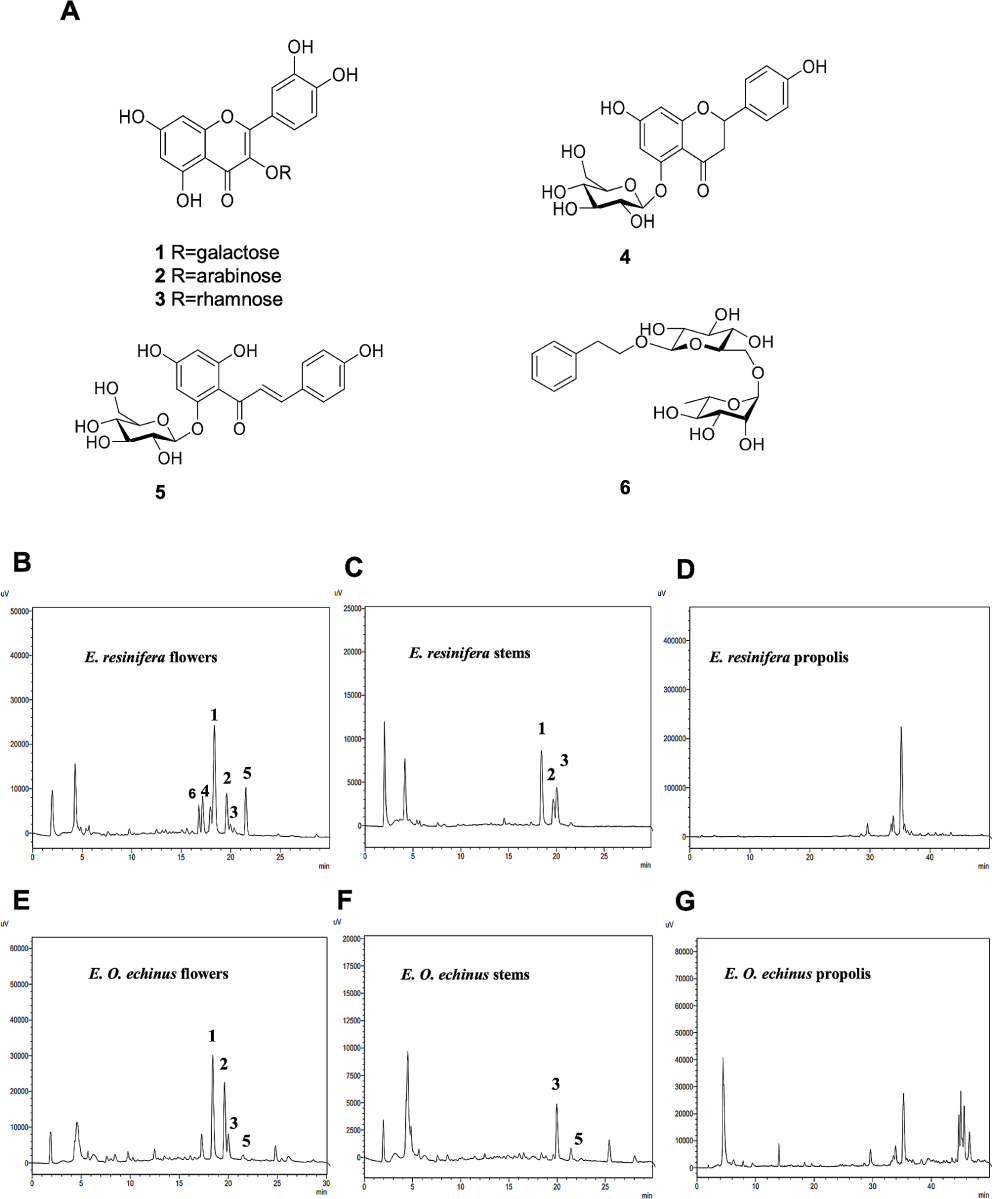
Structural analyses of *Euphorbia* plants. (**A**) Chemical structures of **1**−**6** isolated from ethanol extract of *E. resinifera* flowers. (**B***−***G**) HPLC chromatograms of ethanol extract from *E. resinifera* and *E. echinus* flowers. (1) quercetin-3-*O*-*β*-D-galactospyranoside; (2) quercetin-3-*O*-*β*-D-arabinopyranoside; (3) quercetin-3-*O*-*α* -D-rhamnopyranoside; (4) helichrysin A; (5) isosalipurposide; (6) phenylethyl D-rutinoside

The 70% ethanol extract from the flowers, stems, and propolis of the two *Euphorbia* species was subjected to RP C_18_ Medium-pressure liquid chromatography (MPLC) and High-performance liquid chromatography (HPLC) to yield six known compounds. The compounds were elucidated using 1D and 2D NMR spectroscopy (^1^H, ^13^C, COSY, HSQC, and HMBC) and mass spectrometry. Comparison with the spectroscopic data in the literature determined the compounds as quercetin-3-*O*-*β*-D-galactospyranoside (**1**) [30, 31], quercetin-3-*O*-*β*-D-arabinopyranoside (**2**) [30, 32], quercetin-3-*O*-*α*-D-rhamnopyranoside (**3**) [33], helichrysin A (**4**), isosalipurposide (**5**) [34], and phenylethyl D-rutinoside (**6**) [35] (Fig. 2A). All compounds separated under optimal analysis conditions were analyzed by HPLC. The chromatograms are shown in Fig. 2B-G.

In general, the extract of *E. resinifera* flowers was found to be richer in phenolic compounds than the other extract it has proven to be rich in compounds (1–6), followed by the extract of *E. echinus* flowers with four compounds: (1–3) and (5) The extract containing the least compounds is the extract of *E. echinus* stems; which showed the presence of only two compounds (3) and (5). However, the extract of the stems of *E. resinifera* proved to be rich in compounds (1–3). Regarding propolis extracts, the quantity was too low to identify their compounds.

### Euphorbia extracts inhibit the cell migration of colon cancer cells

**Fig. 3:**
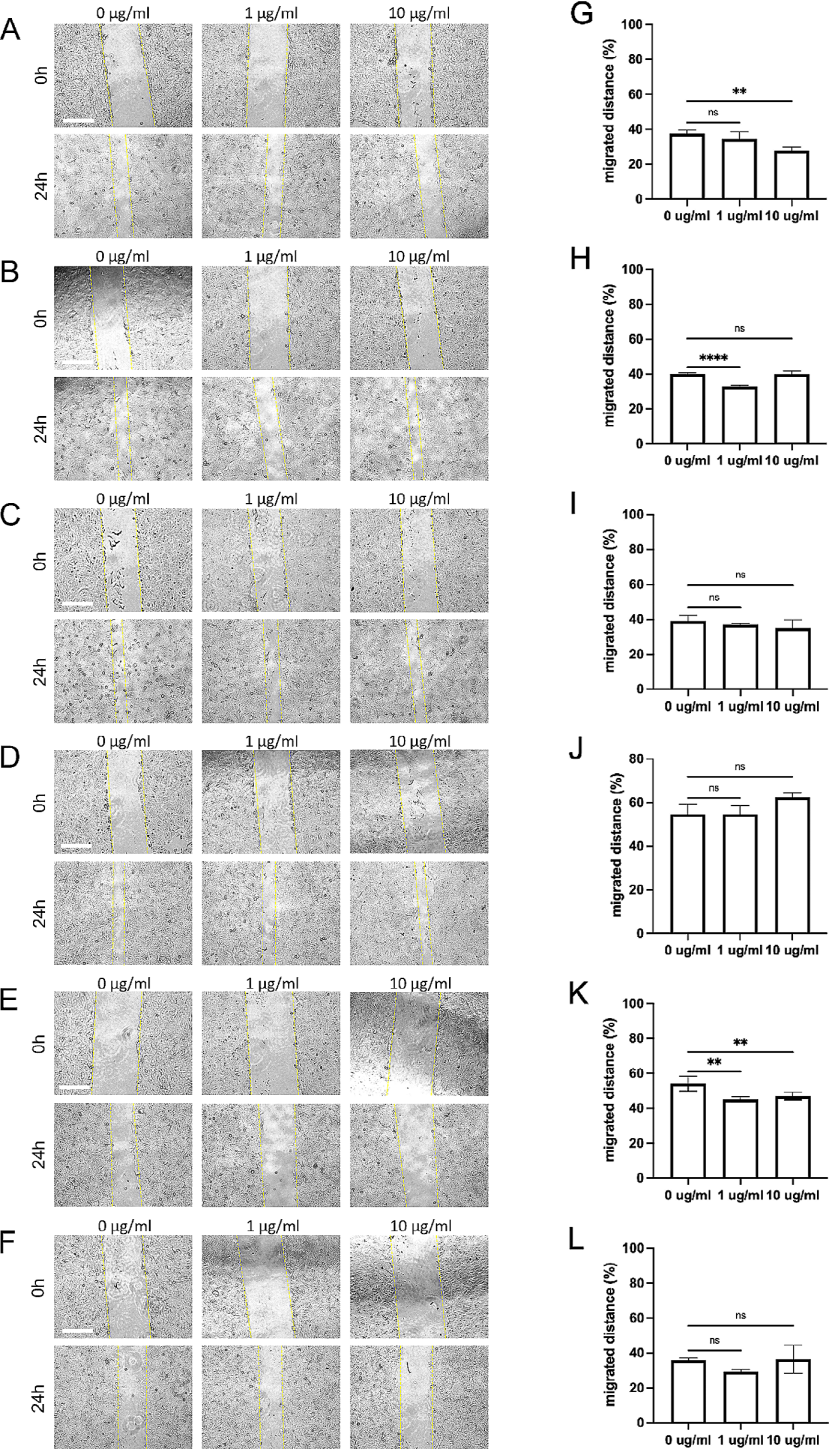
*Euphorbia* extracts inhibit the 24-hour migration of DLD-1 colon cancer cells. The migrated distance percent was measured under a microscope 24 h after the scratches on wells (scale bar = 300 μm) **(A-F)**. Flower petals **(A, D)**, stem **(B, E)**, and propolis **(C, F)** extracts of *E. resinifera* **(A, B, C)** and *E. echinus*  **(D, E, F)** were treated at 0, 1, 10 µg/ml for 24 h. The measured distance 24 h after scratches was compared to time 0 to validate the effect of *Euphorbia* extracts(**G**, *E. resinifera* flower petals;** H**, *E. resinifera* stems; **I**, *E. resinifera* propolis; **J**, *E. echinus* flower petals; **K**, *E. echinus* stems; **L**, *E. echinus* propolis). The measured distance was compared. Data were presented as mean + SD (*n* = 4). *p˂0.05, **p˂0.01, *** p˂0.001, ****p˂0.0001 and ns p˃0.05

Cell migration is a process that plays an essential role in the progression of cancer disease [36]. In vitro-migration assay was performed to determine the potential effect of different plant extracts of the two *Euphorbia* species and their hive products, i.e., propolis, on DLD-1 colorectal cancer cells. The migrated distance of DLD-1 cells was measured before and 24 h after treating *Euphorbia* extracts at 0, 1, and 10 µg/ml (Fig. 3). After treating *Euphorbia* extracts, the migration of DLD-1 cells was significantly inhibited when *E. resinifera* flower extract (Fig. 3A, G) and *E. echinus* stem extract (Fig. 3E, K) were treated. This finding shows that *Euphorbia* extracts have an anti-migratory effect on colon cancer cells.

*E. resinifera* flower extract showed significant inhibition of cell migration as the treated concentration was elevated to 10 µg/ml. Interestingly, the migration of DLD-1 cells was inhibited by *E. resinifera* stem extract at 1 µg/ml, but this inhibition was reverted when the extract was increased to 10 µg/ml. Meanwhile, propolis of *E. resinifera* did not show any significant change in DLD-1 migration.

In the case of *E. echinus*, flower and propolis extracts did not show any significant change in DLD-1 migration. However, the extract from *E. echinus* stem inhibited DLD-1 migration in 1 and 10 µg/ml treated groups.

### Euphorbia flower extracts inhibit cell proliferation of colorectal cancer

**Fig. 4:**
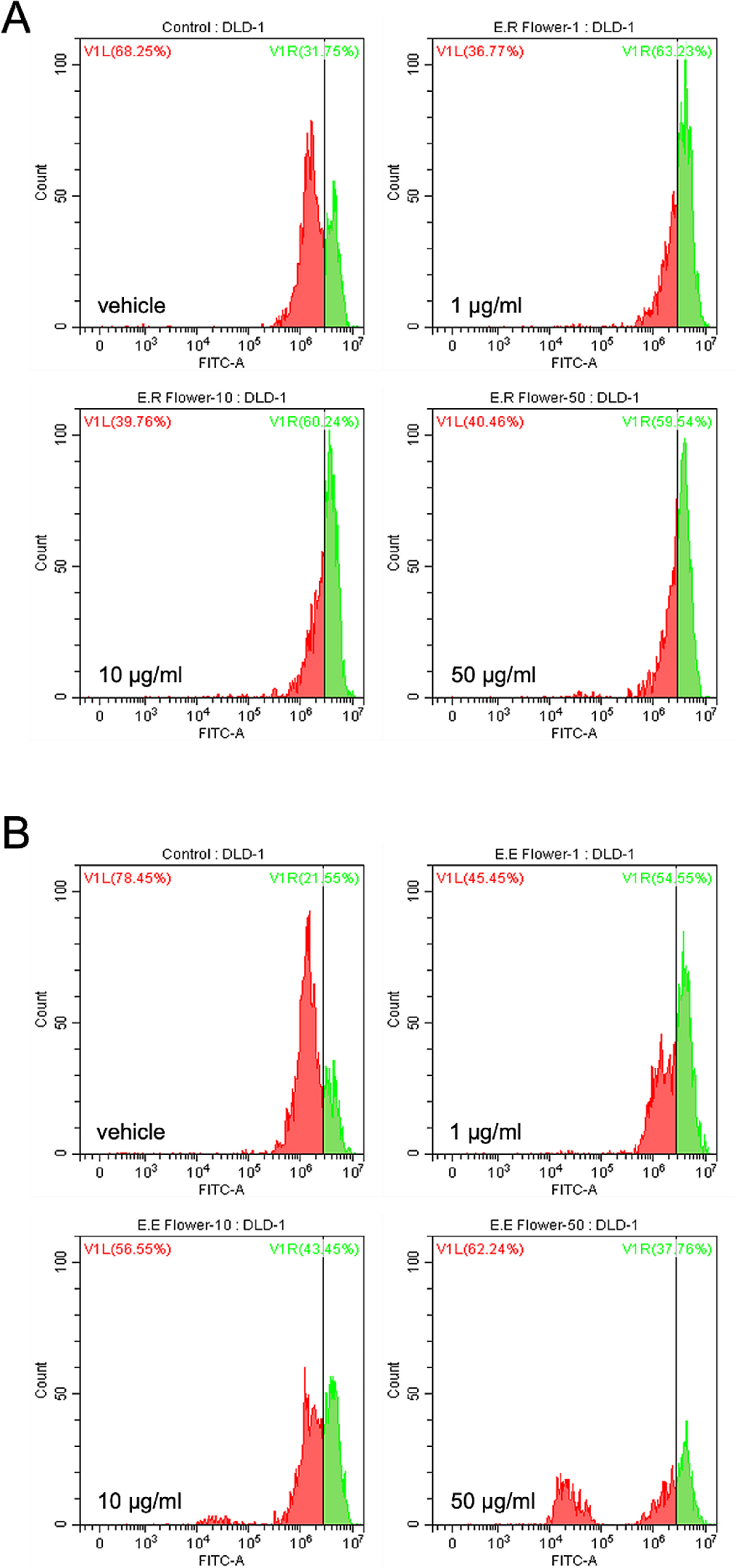
*Euphorbia* flower extracts inhibit the proliferation of colon cancer cells. CFSE-stained DLD-1 cells were analyzed via flow cytometry after 24-hour treatment of flower petal extracts at designated concentrations of *E. resinifera*  **(A)** and *E. echinus*  **(B)** at 0, 1, 10, and 50 µg/ml. The signal of CFSE was measured from 1.0 × 10^5^ events from each group (Blue, non-treated; red, treated)

We assessed the impact of flower extracts, which have proven to be the richest in phenolic compounds for cell proliferation of DLD-1 cells by the CFSE assay. DLD-1 cell lines were strongly affected by treatment with the flower extracts of both *Euphorbia* species after 24 h of treatment (Fig. 4). The flower extract of *E. resinifera* showed more significant inhibition of proliferation, similar for all the different concentrations tested (Fig. 4A).

When the cells were treated with *E. echinus* flower extracts, cell proliferation was generally demonstrated in a dose-dependent manner; a lower concentration of 1 µg/ml showed higher inhibition compared to treatment with the other concentrations (Fig. 4B).

To exclude the toxicity of Euphorbia extracts on normal colon cells, we used FHC cells and measured the proliferation by WST-8 assay. Extracts from both species showed no significant cell death in 1, 10 and 50 µg/ml (Suppl. Figure 1). However, *E. resinifera* propolis showed inhibition of proliferation, which might be an extreme concentration to cells (Suppl. Figure 1B). To show the effect of anti-cancer agents in DLD-1 cells, we treated 0, 0.1 and 1 µM of Osimertinib, which showed no cell death in normal FHC cells (Suppl. Figure 2 A) while DLD-1 showed significant cell death (Suppl. Figure 2B).

### Euphorbia extracts induce apoptosis in colorectal cancer

Apoptosis, the programmed cell death pathway, is an essential process in various biological systems, which occurs during cell aging and development for maintaining homeostasis [37]. To determine the apoptotic effect of flower extracts, we treated the extract on DLD-1 cells for 24 h and then detached cells to propagate to Annexin V/7-AAD stain. A quantitative approach was undertaken to characterize the apoptosis induced by extracts from *E. resinifera* flowers, stems, and propolis, as well as *E. echinus* flower extract, in cell death.

During apoptosis, an early event involves the loss of plasma membrane asymmetry, leading to the translocation of phosphatidylserine (PS) to the outer leaflet of the membrane. Annexin V, a protein that binds to exposed PS, was used to assess this. By conjugating annexin V with FITC (fluorescein isothiocyanate), it becomes detectable by flow cytometry. Annexin V was combined with a vital dye called propidium iodide (PI) to distinguish apoptotic cells from dead cells. Propidium iodide is a nucleic acid intercalator that penetrates the plasma membrane of dead cells.

**Fig. 5:**
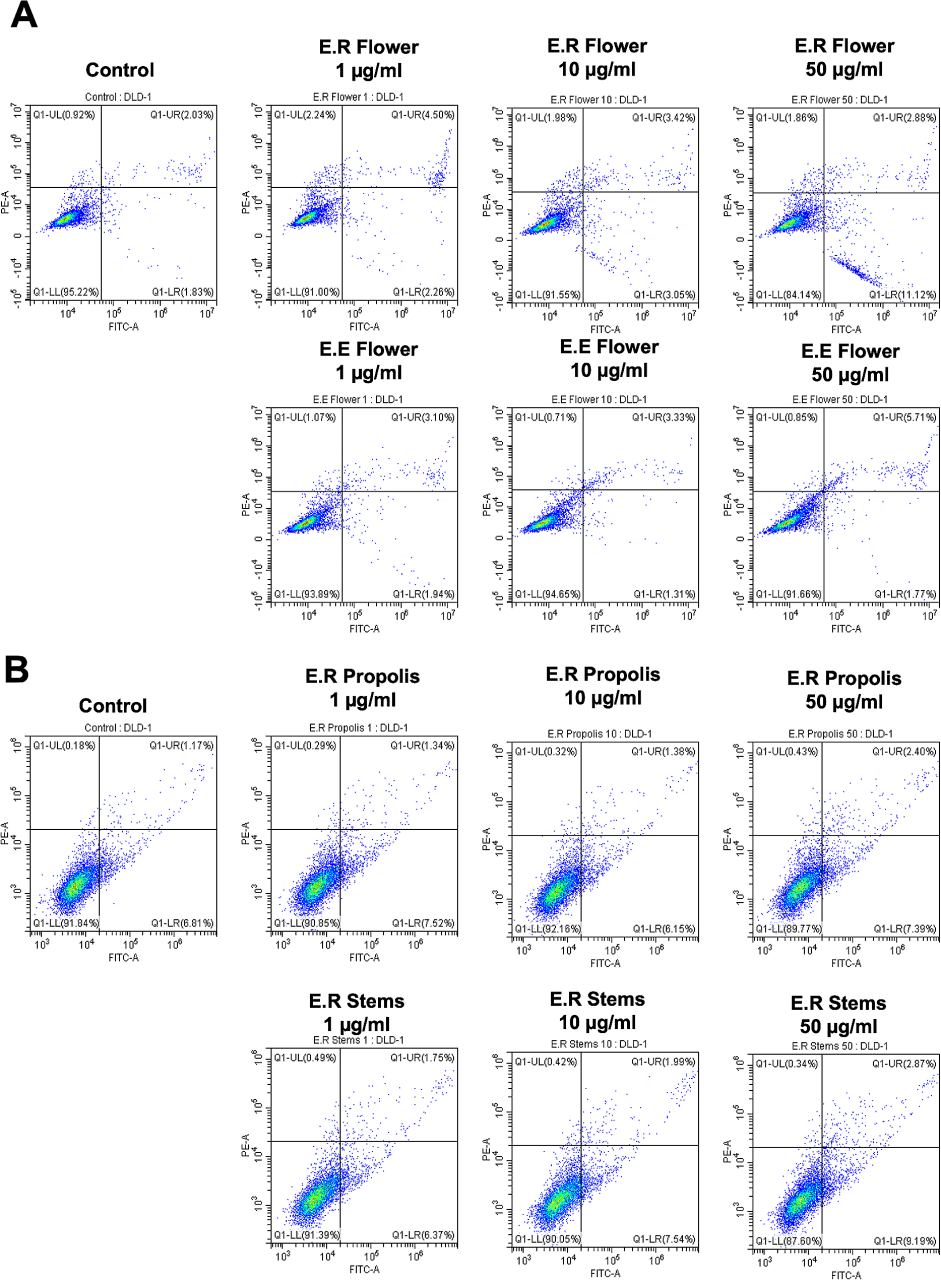
*Euphorbia* extracts induce the apoptotic character of colon cancer cells. Flow cytometry analysis of Annexin V-FITC stained DLD-1 cells after 24 h exposure to 1, 10, and 50 ?g/mL of (**A**) *E. resinifera* (E.R.) flower and *E. echinus* (E.E.) flower and (**B**) *E. resinifera* (E.R.) stem and E. resinifera (E.R.) propolis. Non-apoptotic and non necrotic cells (Annexin V-negative; PI-negative), early apoptotic cells (Annexin V-positive; PI-negative), late apoptotic cells (Annexin V-positive; PI-positive), and necrotic cells (Annexin V-negative; PI-positive) were separately gated to analyze apoptotic events

For the experiment, the cells were incubated with different concentrations of the extracts (0.1, 10, and 50 µg/ml) for 24 h. After the incubation, the cells were stained with annexin V-FITC/propidium iodide and then analyzed using flow cytometry (Fig. 5). After 24 h of incubation, the different concentrations of *E. resinifera* flower extract led to a significant decrease in cell viability. The cell viability decreased from 95.22% in the control group to 84.14% when treated with 50 µg/mL of the flower extract. Moreover, the percentage of early apoptotic cells increased from 1.83% in control to 11.12% at the same concentration of 50 µg/mL (Fig. 5). This dose-dependent increase in early-stage apoptosis suggests the potential apoptotic-inducing properties of *E. resinifera* flower extract.

In contrast, the flower extract of *E. echinus* exhibited a different pattern. Like *E. resinifera*, it also showed a dose-dependent increase in apoptotic cells. However, the effect was more pronounced in late-stage apoptosis. The percentage of cells in late apoptosis increased from 2.03% in control to 5.71% when treated with 50 µg/mL of *E. echinus* flower extract (Fig. 5B).

Additionally, we validated the effects of stem and propolis extracts of *E. resinifera*. When the extracts were treated to DLD-1 cells for 24 h, the apoptotic events of cells were not significantly changed with the treatment of the extracts (Fig. 5C and D).

Osimertinib was treated in DLD-1 cells as the positive control of apoptosis, and the apoptotic events were measured. At a concentration of 0.1µM Osimertinib, cell viability decreased from 73.31% (control) to 67.75%, and at 1µM, it further reduced to 45.76%. Moreover, the percentage of late apoptotic cells increased significantly from 22.49% in the control to 28.06% and 45.29% at 0.1 and 1µM Osimertinib, respectively (Suppl. Figure 3).

These findings highlight the apoptotic effects between *E. resinifera* and *E. echinus* flower extracts. While *E. resinifera* extract induced early apoptosis, *E. echinus* extract led to late apoptosis.

### Mechanistic effects of *Euphorbia* extracts

The expression levels of numerous proteins related to the anti-cancer effect were ascertained within DLD-1 cells to elucidate potential mechanisms inherent to extracts derived from two distinct *Euphorbia* species.

**Fig. 6:**
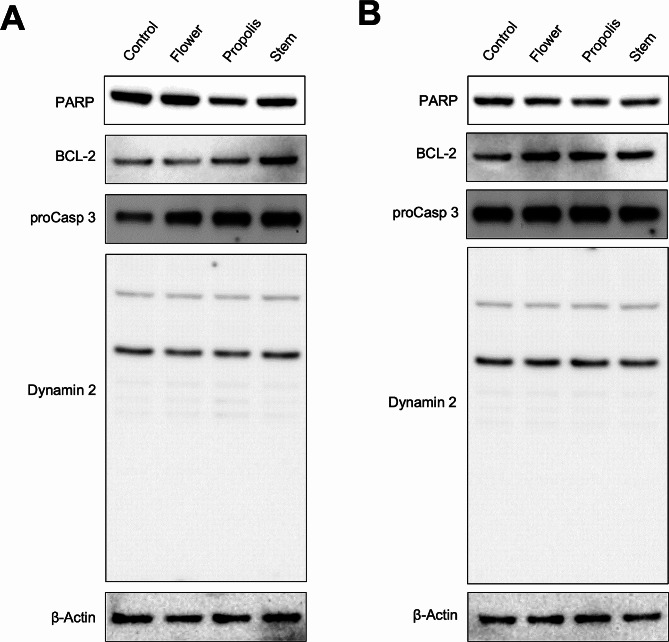
*Euphorbia* extracts show anti-cancer effects at molecular levels. Western blot analyses of DLD-1 cells. Cells were grown in 6-well plates overnight and then treated with *E. echinus* flower petals, stem, or propolis **(A)** and *E. resinifera* flower petals, stem, or propolis **(B)** at 0, 10 µg/ml for 24 h. The protein expression levels of PAPR, Bcl-2, Caspase-3, and Dynamin-2 were detected from the membrane. The raw data of PARP, Bcl-2 and Caspase-3 is shown in Supplementary Fig. 4

To measure the anti-cancer effects in molecular levels, PARP, Bcl-2, Caspase-3, and Dynamin-2 were measured from the cell lysate treated with *Euphorbia* extracts. Propolis and stem extracts of *E. echinus* increase Caspase-3 expression compared to the control. Expression of Bcl-2 is also increased under the same conditions. Conversely, compared to the control, PARP expression is diminished in propolis and stem extracts. In the case of the flower extract, a notable rise in Caspase-3 expression is observed, while the levels of Bcl-2 and PARP expression do not exhibit significant changes compared to the control (Fig. 6A).

For *E.resinifer*, the stem extract lacks significant change in Caspase-3 expression, which indicates that this specific pathway might not have been prominently activated by the extract and stem extract could be operating in a manner independent of the classical caspase-dependent apoptotic pathway **(**Fig. 6B**)**.

The reduction in PARP might reveal that cancer cells struggle to repair their DNA, possibly leading to cellular stress signals and eventual activation of apoptosis through an alternative pathway. On the other hand, the increase in Bcl-2 suggests that the extract might counter apoptosis by preserving mitochondrial membrane integrity and blocking the release of pro-apoptotic molecules.

Meanwhile, the migration marker of cancer cells, Dynamin-2, does not show significant change with the treatment of the extracts of both species. These observations match well with the subtle migration change mentioned in Fig. 3.

## Discussion

Knowing the constituent molecules present in the extracts of the flowers, stems, and propolis of both species has the possible usage in therapy. This is particularly significant considering that the profile of phenolic compounds in these extracts is being examined for the first time. To achieve this, we conducted High-performance liquid chromatography (HPLC) HPLC analysis on the ethanolic extracts of all the plant parts. Selecting an appropriate extraction solvent is crucial in isolating target compounds from plant materials. In this study, 70% ethanol was chosen as the extraction solvent for the two Euphorbia species’ flowers, stems, and propolis. Selecting an appropriate extraction solvent is crucial in isolating target compounds from plant materials. In this study, 70% ethanol was chosen as the extraction solvent for the two Euphorbia species’ flowers, stems, and propolis.

In previous studies, the choice of 70% ethanol as the extraction solvent is justified by observing higher concentrations of bioactive flavonoid compounds in the ethanol extracts. This can be attributed to the increased polarity from adding water to pure ethanol. By incorporating 30% water to prepare the 70% ethanol solution, the overall polarity of the solvent was effectively improved [38, 39]. Additionally, the researchers discovered that ethanol, being less polar than water, facilitated the release of total polyphenols from cell walls due to its effectiveness in degrading the non-polar character of the cell walls. As a result, higher values of total polyphenols were observed in the ethanol extract compared to the water extract, as reported in a study by Bimakr et al. in 2010 [40]. The flower extracts of the plants exhibited a higher abundance of phenolic compounds when compared to the other extracts.

The extract obtained from *E. resinifera* flowers contained six compounds, including quercetin-3-O-β-D-galactospyranoside, quercetin-3-O-β-D-arabinopyranoside, quercetin-3-O-α-D-rhamnopyranoside, helichrysin A, isosalipurposide, and phenylethyl D-rutinoside. In comparison, the extract of *E. echinus* flowers contained four compounds, namely quercetin-3-O-β-D-galactospyranoside, quercetin-3-O-β-D-arabinopyranoside, quercetin-3-O-α-D-rhamnopyranoside, and isosalipurposide. These findings align with previous studies that have identified flavonoids, particularly quercetin glycosides, in various above-ground parts of *Euphorbia* species [41, 42]. The Flavonoid family has been reported to exhibit anti-proliferative activity [43] and can induce apoptosis when combined with its derivatives [44]. Several studies have investigated the effects of quercetin obtained from specialized suppliers, demonstrating its anti-proliferative and apoptosis-inducing effects on human breast cancer cells (MDA-MB-453) [45]. Additionally, research by Refolo, M.G. et al. confirmed that quercetin specifically affects the proliferation and migration of colon cancer cells (Caco2 cells) [46]. Moreover, quercetin isolated from apple peels has shown potent anti-proliferative activity against HepG2 and MCF-7 cells [47]. Furthermore, quercetin-3-O-β-D-galactopyranoside has been found to possess anti-cancer activity against skin cancer cell lines through various mechanisms, including regulating proliferation, autophagy, and apoptosis [48]. Additionally, isosalipurposide, isolated from the ethanolic extract of Helichrysum maracandicum flowers, has been reported to have anticarcinogenic activity [49]. However, no Helichrysin A and phenylethyl D-rutinoside reports exhibited any anti-cancer properties. The same compounds we detected in our extract may be responsible for the observed anti-proliferative activity in DLD-1 cells.

*Euphorbia resinifera O. Berg* (*E. resinifera*) and *Euphorbia officinarum subsp. echinus (Hook. f. & Coss.) Vindt* (*E. echinus*), two species from the Euphorbiaceae family, have a rich history of traditional use among the local Moroccan population to treat colon cancer [50].

Firstly, we determined whether the extract of Euphorbia affected both colon cancer cells and normal cells. Various techniques have been utilized when examining cell viability analyses presented in the literature. These include assays such as WST-8, MTT, Alamar Blue, cell cycle analysis, neutral red release assay, and trypan blue exclusion assay. However, it’s important to note that these diverse methods do not consistently produce similar results. [51–54]. The observations shed light on divergent cellular responses depending on the cell and extract types. Euphorbia extracts do not exhibit apparent toxicity towards DLD-1 cells and even induce enhanced cell viability under most conditions, while FHC cells are unaffected. These results concerning the DLD-1 cells can be elucidated by the fact that the WST-8 assay quantifies the conversion of the reagent into formazan, which does not directly gauge cell viability. Viable yet non-metabolically active cells may yield falsely negative outcomes [55].

In this study, we employed a range of assays to investigate and compare the mechanisms underlying the anti-cancer effects of these plants on human DLD-1 colon cancer cells in an in vitro setting. Specifically, we explored the impacts of extracts derived from various plant parts of *E. resinifera* and *E. echinus* and bee products such as propolis on cell migration, proliferation, and apoptosis. Several studies have documented the cytotoxic properties of *Euphorbia* latex or whole plants from specific species within the *Euphorbia* genus [50, 56–60]. However, there is a dearth of research examining the specific contributions of different parts of *Euphorbia* plants, specifically *E. echinus* and *E. resinifera*, in the context of cancer studies. Tumor metastases are the leading cause of cancer death. Colorectal cancer is the third most common cancer [61], and it was reported that approximately 20% of patients with colorectal cancer had been reported to present metastases at diagnosis, which can decrease the survival rate from about 56% of patients from 5 years to low survival rate [62]. The crucial roles of cell migration in cancer cell dissemination, tissue invasion, and metastasis have been extensively examined by numerous experts in the field of cancer research. Through their investigations, these researchers have provided valuable insights into the development of malignant tumors (cancers) and the underlying mechanisms involved in tumor progression [63].

### **In the bibliography, there is a lack of studies addressing the anti-migration activity of extracts from***E. resinifera***and***E. echinus***on cancer cells**

Our results demonstrated that both species’ flower, propolis, and stem extracts tend to inhibit DLD-1 cell migration. The extracts that gave significant results after 24 h were the *E. resinifera* flower extract and *E. echinus* stem extract, which inhibited the migration of DLD-1 cells by as much as 10%. These findings suggest that these extracts possess anti-migratory properties, which can impede the progression and metastasis of colon cancer. Interestingly, the inhibitory effect of *E. resinifera* flower extract on cell migration became more pronounced as the concentration increased to 10 µg/ml. In contrast, the inhibition observed with *E. resinifera* stem extract at 1 µg/ml was diminished when the concentration was raised to 10 µg/ml. These observations indicate a potential dose-dependent effect of the extracts on cell migration. However, the stem extract of *E. echinus* demonstrated inhibitory effects on cell migration at both 1 µg/ml and 10 µg/ml treated concentrations. This highlights the potential anti-migratory properties associated specifically with the stem extract of *E. echinus*.

Later, we selected flower extracts of the two plants to analyze the anti-proliferative effect by the CFSE assay, which showed the ability to inhibit the proliferation of DLD-1 cells.

No previous data were found on the effects of *E. resinifera* and *E. echinus* extracts in human colon cancer cells, and the studies about the colon cancer effects of herbal extracts on colorectal cancers were limited. Previous reports by other researchers have documented the anti-migration of other *Euphorbia* species, including the hydroalcoholic extract of *E. lacteal*, which showed maximum inhibition (about 40%) at the concentration of 125 µg/ml against the migration of HN22 cells [64]. Another study on an ethanolic extract from seeds of *E. lathyris* showed a reduction in the migration capacity of 18.68% after 72 h of colon cancer cells (T84 cell line) [65]. In the same context, a study was made on propolis obtained from northern China, which showed that the ethanolic extract of this propolis showed significantly reduced cell migration at a concentration (25, 50, and 100 µg/ml) after 24 and 48 h on HepG2 hepatocellular carcinoma cells [66].

The apoptosis mechanism is one of the biomedical strategies for cancer treatment in the boundary of chemotherapy and chemoprevention [67]. In this study, Annexin V/7-AAD assay was performed to prove the mode of cell death induced by *E. resinifera* propolis, *E. resinifera* stem, *E. resinifera* flower, and *E. echinus* flower extracts, which showed significant and remarkable induction of DLD-1 apoptosis. Based on our results, *Euphorbia* extracts induced apoptosis in this cell line. The extract from the *E. resinifera* flowers shows a high ability to enhance apoptosis-mediated cell death. Therefore, the extract is considered promising as a potential anti-cancer candidate without significant toxicity to normal colon cells. So far, many plants and medicinal compounds have shown anti-cancer effects by inducing apoptosis.

After assessing the evaluation of various *Euphorbia* extracts for their anti-proliferative and apoptotic impacts, we unraveled the fundamental molecular mechanism responsible for initiating apoptosis. This was achieved by examining the expression of apoptosis-related proteins such as caspase 3, PARP, and Bcl-2.

The pivotal biochemical event central to the process of apoptosis was the increase in pro-apoptotic proteins and/or the decrease in molecules of anti-apoptotic proteins.

Caspase-3, a vital component in the execution of apoptosis, represents the active configuration procaspase-3. Activation of caspase-3 can arise from both the intrinsic pathway initiated by mitochondria and the extrinsic pathway activated by death receptors [68]. This, in turn, sets in motion various apoptotic processes downstream, resulting in characteristic biochemical and morphological changes within the cell. Based on the findings of this research, the increased susceptibility of DLD-1 cells to apoptosis when treated with various *Euphorbia* extracts notably aligns with the concurrent rise in caspase-3 activity, except for *E. resinifera* stem extract.

PARP, functioning as a DNA repair enzyme, becomes active in response to DNA breaks, facilitating the addition of ADP-ribose polymers to different nuclear components to accelerate repair processes. PARP holds significance for cell survival. However, the cleavage of PARP contributes to cellular breakdown and indicates cells undergoing apoptosis. In apoptosis, caspases trigger the cleavage and deactivation of PARP [69]. The decrease in PARP expression implies that DLD-1 cells treated with Euphorbia extracts could encounter disturbances in DNA repair mechanisms, thus facilitating the initiation of apoptosis. The Bcl-2 protein is an anti-apoptotic molecule, which raises its expression and causes a delay in disrupting organelles surviving cells from apoptosis [70]. These proteins are found on the outer mitochondrial membrane and play a particularly crucial role in the intrinsic apoptotic pathway [71]. The stable expression of Bcl-2 in cells treated with extracts from *E. echinus* flowers could suggest that this anti-apoptotic protein has not been directly affected by the ongoing anti-cancer treatment. This might imply that the cancer cells have maintained their resistance to apoptosis through a consistent Bcl-2 expression. Conversely, the increased expression of Bcl-2 in other extracts could be explained by cancer cells attempting to counteract the treatment’s effects by enhancing their resistance to apoptosis. This could also indicate an adaptive response of cancer cells to the pressure exerted by the anti-cancer treatment. Also, the observation that the expression of Dyanmin-2 remains relatively unchanged across all tested extracts compared to the control group also sparks intrigue. This suggests that the apoptotic effects induced by these extracts might occur independently of Dyanmin-2 regulation. As such, these extracts may potentially target key apoptotic pathways that exhibit limited dependence on Dyanmin-2.

In summary, this study provides an enlightening perspective on the distinct mechanisms underpinning the apoptotic effects of Euphorbia extracts. The intricate alterations in the expression of pivotal proteins hint at sophisticated interactions and the potential activation of alternative apoptotic pathways. These findings deepen our comprehension of the intricate regulatory mechanisms governing cellular fates, paving the way for promising research in anti-cancer therapy and cellular biology.

Our research focused exclusively on two species of *Euphorbia*, namely *E. resinifera* and *E. echinus*. As a result, generalizing the findings to other species or different types of cancer may present challenges. Conducting a broader study encompassing a wider variety of plant species and cancer types would provide a more comprehensive understanding of the anti-cancer activity of these plant extracts. Furthermore, our research primarily centered on in vitro studies. Assessing the effectiveness of plant extracts in contexts closer to reality, such as in animal models or clinical studies, would provide valuable insights and a better understanding of their therapeutic potential for patients with colon cancer.

Exploring potential interactions between *Euphorbia* plant extracts and commonly used anti-cancer agents is an intriguing avenue for future research. Combination studies could uncover synergistic or complementary effects, thereby enhancing the overall efficacy of treatment approaches, as many cancer therapies already employ combination strategies.

In addition, conducting pharmacokinetic studies on *Euphorbia* plant extracts would yield valuable information regarding their absorption, distribution, metabolism, and elimination within the body. Such data is essential for determining appropriate concentrations and dosing regimens for subsequent clinical studies. Addressing these limitations and pursuing these perspectives would contribute to a more comprehensive understanding of the anti-cancer potential of *Euphorbia* plant extracts and pave the way for their effective utilization in cancer treatment strategies.

## Conclusions

The *Euphorbia* flower extract emerges as a highly promising candidate for anti-cancer therapy, as it has demonstrated, for the first time, remarkable potential in inhibiting the growth of DLD-1 colon cancer cells. Our study revealed that these flower extracts possess the capability to induce apoptosis, a vital mechanism in combating cancer. These findings strongly suggest that the flower extracts from both *Euphorbia* species harbor bioactive compounds that contribute to their anti-cancer activity.

### Electronic supplementary material

Below is the link to the electronic supplementary material.

Supplementary Material 1

Supplementary Material 2

Supplementary Material 3

Supplementary Material 4

## Data Availability

The datasets used and/or analyzed during the current study are available from the corresponding author upon reasonable request.
