# Supplementary material for: Two spurge species, Euphorbia resinifera O. Berg and Euphorbia officinarum subsp. echinus (Hook.f. & Coss.) Vindt inhibit colon cancer
Source: BMC Complement Med Ther. 2024 Jul 10;24:261. doi: 10.1186/s12906-024-04566-3 (PMC11238497; doi:10.1186/s12906-024-04566-3)
Supplement: Supplementary file 1 — Supplementary Material 1 [file 12906_2024_4566_MOESM1_ESM.pdf]

Supplementary Figure 1

A

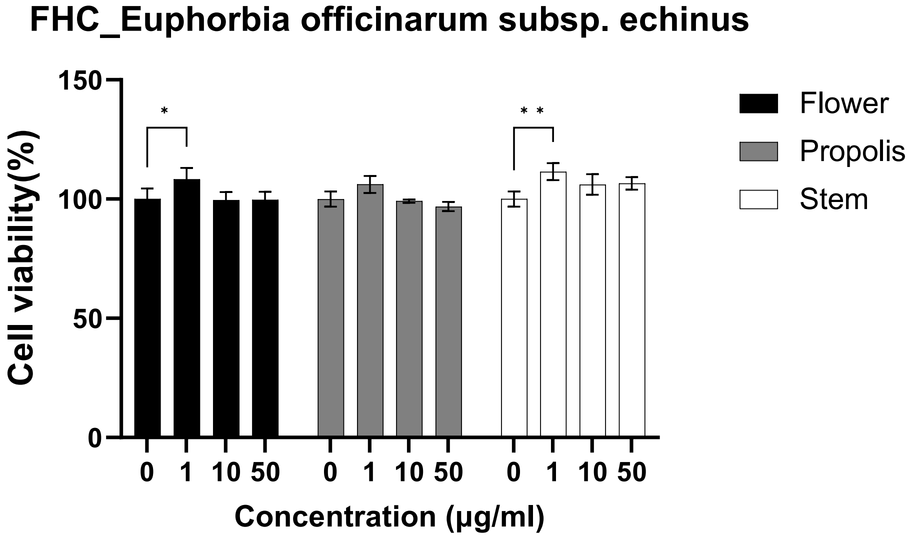

B

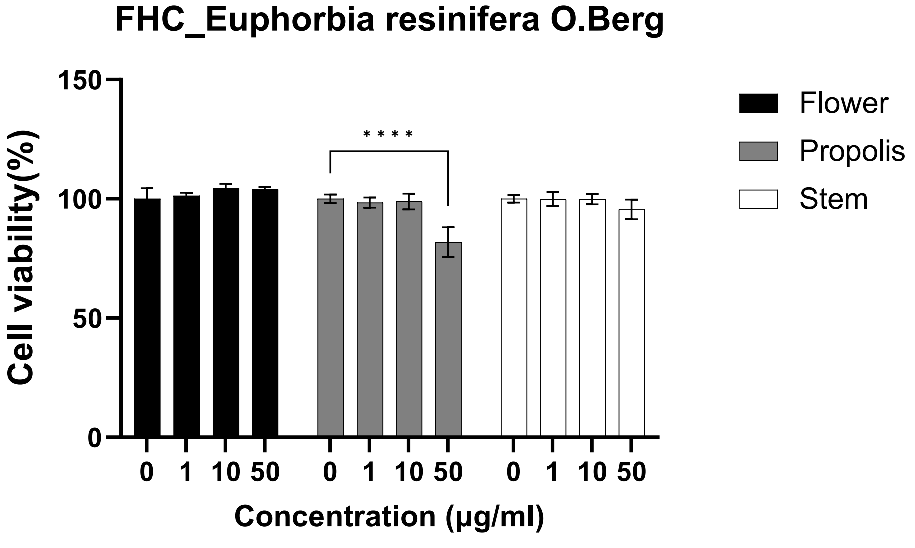

WST-8 cell viability assay. FHC cells were treated with *E. echinus* flower petals, stem, or propolis (A) and *E. resinifera* flower petals, stem, or propolis (B) at 0, 1, 10, and 50 μg/ml for 24 hours.
