# Supplementary material for: Two spurge species, Euphorbia resinifera O. Berg and Euphorbia officinarum subsp. echinus (Hook.f. & Coss.) Vindt inhibit colon cancer
Source: BMC Complement Med Ther. 2024 Jul 10;24:261. doi: 10.1186/s12906-024-04566-3 (PMC11238497; doi:10.1186/s12906-024-04566-3)
Supplement: Supplementary file 2 — Supplementary Material 2 [file 12906_2024_4566_MOESM2_ESM.pdf]

Supplementary Figure 2

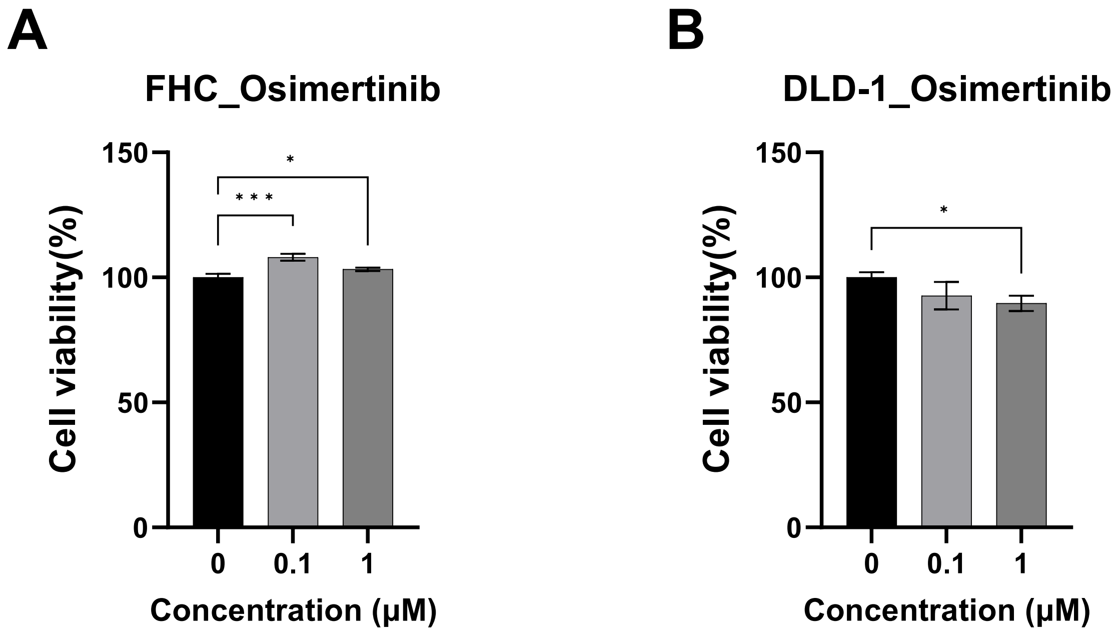

WST-8 cell viability assay. FHC cells or DLD-1 cells were treated with Osimertinib at 0.1, 1 μg/ml for 24 hours.
