# Supplementary material for: Two spurge species, Euphorbia resinifera O. Berg and Euphorbia officinarum subsp. echinus (Hook.f. & Coss.) Vindt inhibit colon cancer
Source: BMC Complement Med Ther. 2024 Jul 10;24:261. doi: 10.1186/s12906-024-04566-3 (PMC11238497; doi:10.1186/s12906-024-04566-3)
Supplement: Supplementary file 3 — Supplementary Material 3 [file 12906_2024_4566_MOESM3_ESM.pdf]

Supplementary Figure 3

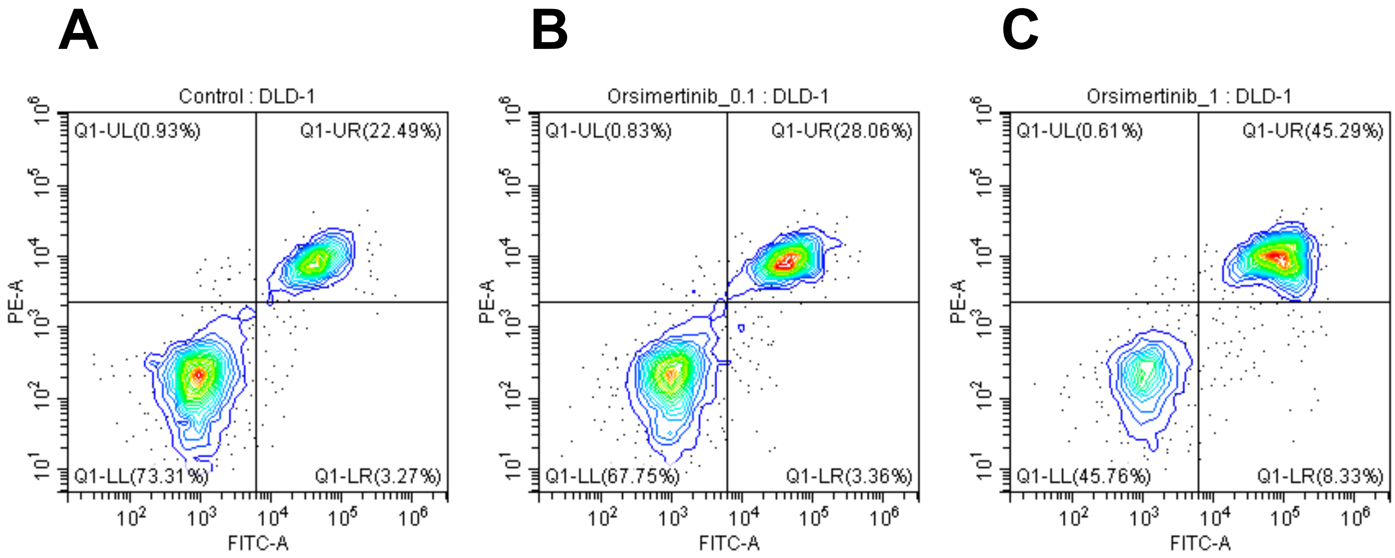

Flow cytometry analysis of PI-Annexin V-FITC stained DLD-1 cells after 24 h exposure to 0 (A), 0.1 μM (B), 1 μM (C) of Osimertinib. Normal cells (Annexin V-negative; PI-negative), early apoptotic cells (Annexin V-positive; PI-negative), late apoptotic cells (Annexin V-positive; PI-positive), and necrotic cells (Annexin V-negative; PI-positive) were separately gated to analyze apoptotic events.
