# Supplementary figures and images for: Two spurge species, Euphorbia resinifera O. Berg and Euphorbia officinarum subsp. echinus (Hook.f. & Coss.) Vindt inhibit colon cancer
Source: BMC Complement Med Ther. 2024 Jul 10;24:261. doi: 10.1186/s12906-024-04566-3 (PMC11238497; doi:10.1186/s12906-024-04566-3)

## Supplementary Figure 4

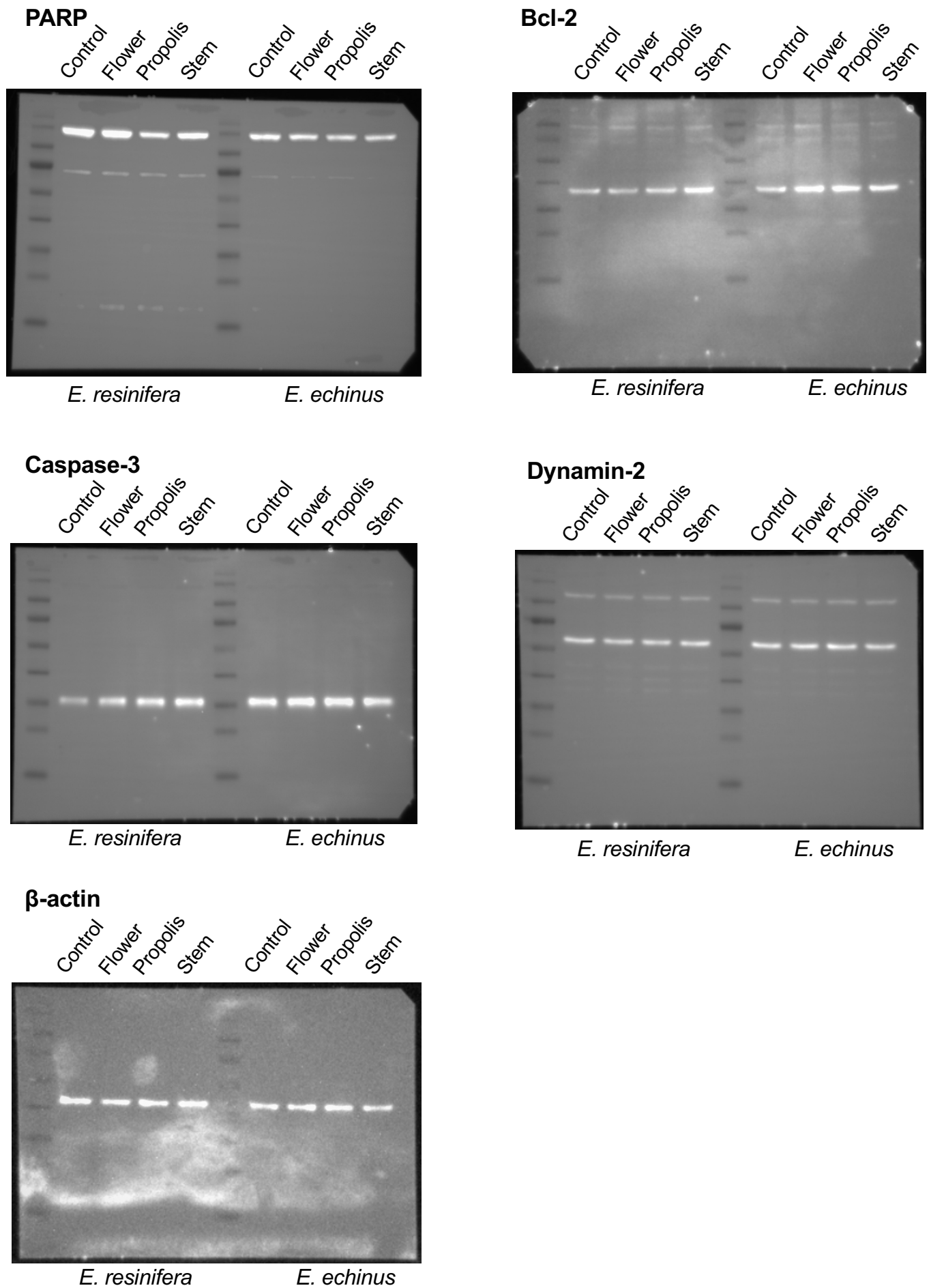

The raw data of western blot images in Figure 6 is shown.

Supplement: Supplementary file 4 — Supplementary Material 4 [file 12906_2024_4566_MOESM4_ESM.pdf]
